# Co-existing tuberculosis and malignant mesothelioma with multiple sites venous thrombosis: a case report

**DOI:** 10.1186/s13104-016-2215-z

**Published:** 2016-08-19

**Authors:** Pratyush Kumar, Kunal Chawla, Pooja Khosla, Sunil Jain

**Affiliations:** 1 Department of Family Medicine, Sir Gangaram Hospital, New Delhi, 110060 India; 2 Department of Medicine, Sir Gangaram Hospital, New Delhi, India

**Keywords:** Case report, Tuberculosis, Malignant mesothelioma, Venous thrombosis, Chylothorax

## Abstract

**Background:**

Tuberculosis is endemic in India and almost 40 % of the Indian population is infected with tubercle bacilli. Tuberculosis being a great mimicker of infectious as well as non infectious diseases and recent rise of multi drug resistant and extended drug resistant cases have made diagnosis and management more difficult. To the best of our knowledge there have been no reported cases of tuberculosis coexisting with malignant peritoneal mesothelioma leading to multiple site venous thrombosis.

**Case presentation:**

Forty five year old male, belonging to Indian/Aryan ethnicity presented with cough, breathlessness and fever for 7 months with past history of pulmonary tuberculosis. On examination he was found to have pleural effusion for which he received anti-tuberculosis therapy empirically. Later his condition deteriorated and on further examination he was found to have ascites, multiple site venous thrombosis and pyothorax which was found positive for acid fast bacilli. Despite anti-tuberculosis therapy he did not improve and was suspected to be a multidrug resistant case. Later on computed tomography peritoneal nodule was detected and on biopsy revealed malignant mesothelioma.

**Conclusion:**

In a diagnosed case of tuberculosis with clinical findings compatible with it but not responding to anti tubercular therapy, underlying secondary co-existing pathology should be explored.

## Background

Tuberculosis (TB) is considered as a great mimicker of infectious as well as non infectious pathology with multitude of clinical picture and variations. Its ability to infect virtually any organ makes it a real health care challenge in the developing world [1]. It is estimated that almost 40 % of the Indian population is infected with tubercle bacilli [2]. A poor response to treatment in a diagnosed case of tuberculosis usually indicates drug resistant tuberculosis, however rarely it can point towards another underlying illness which has remained undiagnosed. More virulent and resistant strains leading to multi drug resistant and extended drug resistant tuberculosis have made it extremely difficult to diagnose a co infection or coexisting pathology.

Here we report a rare case of extra pulmonary tuberculosis with acid fast bacilli positive in pleural fluid and multiple sites of venous thrombosis with poor response to anti-tuberculosis therapy (ATT). Subsequent evaluation revealed an underlying peritoneal mesothelioma.

## Case presentation

A 45 year old male of Indian/Aryan ethnicity who was apparently normal 7 months back was referred to our hospital with complaints of cough for 7 months which was dry in nature and associated with breathlessness and fever. He had a past history of pulmonary tuberculosis, 20 years ago for which he received anti-tuberculosis therapy for a period of 12 months. Three years ago he also developed cholelithiasis for which he underwent cholecystectomy.

He was evaluated elsewhere and on basis of chest X-ray findings of consolidation and pleural effusion, anti-tuberculosis therapy (rifampicin, isoniazid, ethambutol and pyrazinamide) was started. Inspite of 3 months therapy his symptoms did not subside and he started noticing gradual abdominal distension. He was shifted to a tertiary care centre in Dehradun where he was found to have bilateral pleural effusion with ascites. Thoracocentesis was done which showed cell count—30 (polymorph 10 % and lymphocytes 90 %), glucose—92 mg/dl, protein—2800 mg/dl, adenosine deaminase—0.93. Cartridge-based nucleic acid amplification test for tuberculosis was negative. Contrast enhanced computed tomography (CT) revealed gross ascites, bilateral pleural effusion, mild pericardial effusion and inferior vena cava thrombosis. He was then referred to another tertiary care centre at Delhi and he was re-evaluated. Intercostal drainage (ICD) tube was inserted and pus drained from the pleural cavity which was positive for acid fast bacilli (AFB) on Ziehl–Neelsen stain. Fine needle aspiration and cytology of right axillary lymphnode showed reactive lymphadenitis. Repeat CT of chest and abdomen revealed similar finding as before with additional thrombosis in superior and inferior vena cava, right internal jugular vein and bilateral brachiocephalic veins.

Patient was then brought to our hospital. On examination he was conscious and oriented. Pallor and anasarca were present. Bilateral crepitations with decreased air entry at base and right sided ICD tube were noted. His abdomen was distended and shifting dullness was present.

Hematological profile revealed microcytic hypochromic anemia with mild leukocytosis. Urine routine microscopy revealed proteinuria. Renal function tests were normal, liver function tests revealed hypoalbuminemia. Blood culture and urine culture were sterile and procalcitonin was 0.07. Anti nuclear antibody and lupus anticoagulant were negative.

**Fig. 1:**
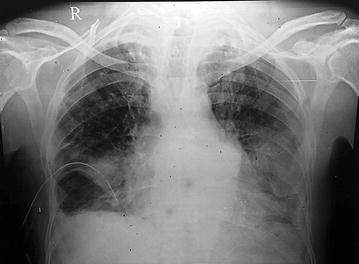
Chest x-ray showing ill defined radio opacity in *left lower zone*, fibrotic opacities with subsegmental atelectasis seen in bilateral *upper zones* and bilateral pleural effusion with ICD tube in situ on the *right side*

Chest x-ray revealed ill defined radio opacity in left lower zone, fibrotic opacities with subsegmental atelectasis seen in bilateral upper zones and bilateral pleural effusion with ICD tube in situ on the right side (Fig. 1).

Thoracocentesis was done and chylous fluid was aspirated. Laboratory evaluation of the fluid revealed cell count—38 polymorphs 10 % and lymphocytes 90 %, protein—1.1 gm/dl, albumin—0.7, glucose—116, ADA 4.0, triglycerides −360 mg/dl, cholesterol—36 mg/dl, culture-sterile, KOH stain-negative, gram stain-negative. Cytology was negative for cancer and consistent with chylothorax.

**Fig. 2:**
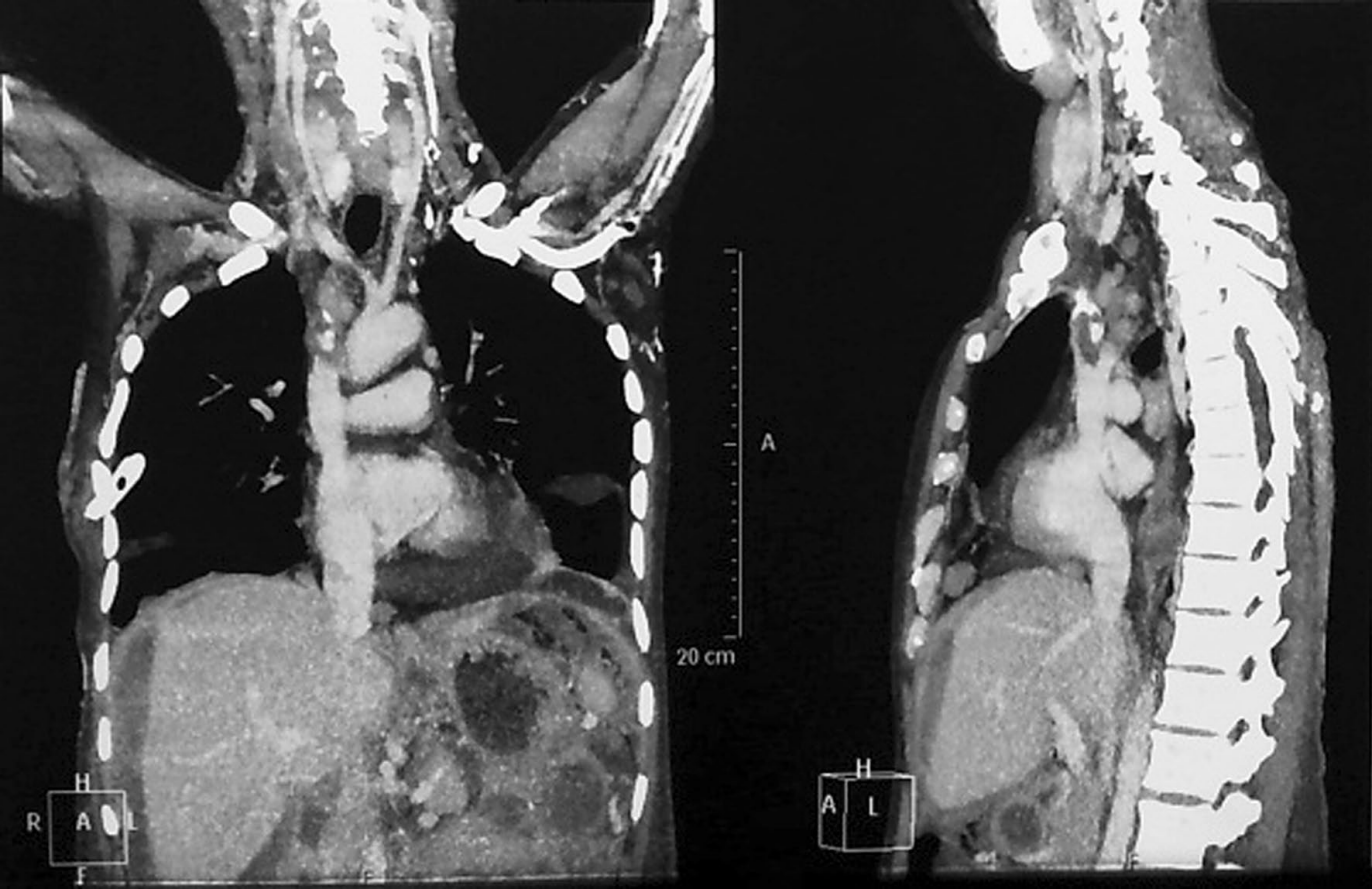
CT venography chest and abdomen showing hypodense superior vena cava and infra renal inferior vena cava suggestive of thrombosis with mid ascites with omental thickening

**Fig. 3:**
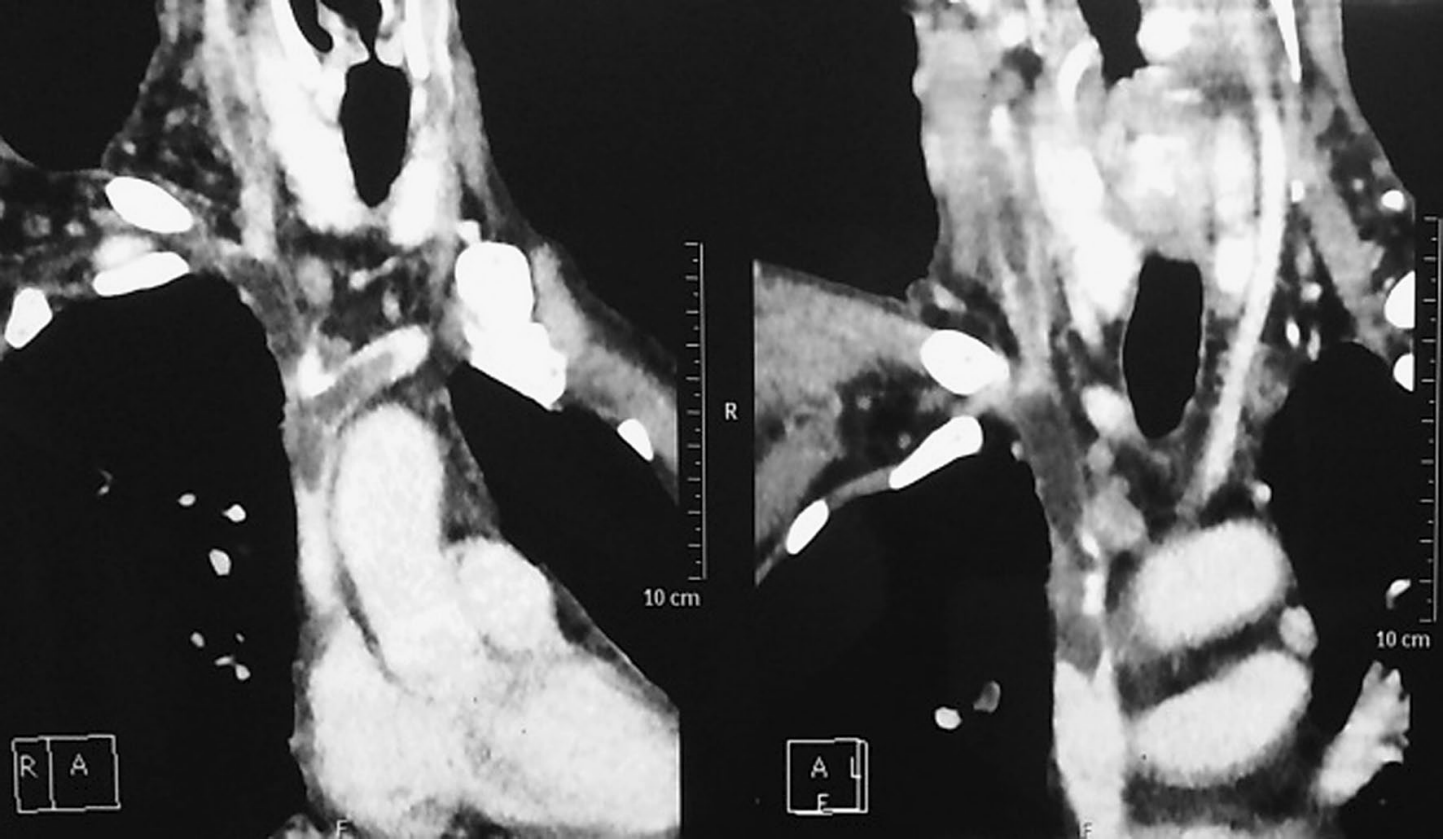
CT venography chest showing evidence of pulmonary thromboembolism and hypodense filling defect in the bilateral brachiocephalic veins *right* internal jugular vein

CT venography chest and abdomen was done which showed hypodense filling defect in the bilateral brachiocephalic veins, right internal jugular vein, superior vena cava and infra renal inferior vena cava suggestive of thrombosis. Pulmonary thromboembolism was also noted. (Figs. 2, 3) mild ascites with omental thickening with diffuse wall thickening of small bowel was noted.

In view of these findings, omental biopsies along with subdiaphragmatic lymph node biopsy were done. Histopathology showed malignant mesothelioma with nodal metastasis. Under oncology care low dose chemotherapy including carboplatin and pemetrexed were given.

He withstood the first cycle of chemotherapy well and gradually his anasarca reduced. Daily regimen of four drugs ATT (rifampicin, isoniazid, ethambutol and pyrazinamide) was also continued and he was discharged in a clinically stable condition with advice to follow-up for chemotherapy. He is now on regular follow-up with medical oncologist.

## Discussion

Tuberculosis in India is an ancient disease with reference dating as far back as 1500 BC [3]. There is huge burden of tuberculosis with annual mortality of more than 300,000 nationwide [2]. Tubercular pleural effusion, earlier thought to be a hypersensitive reaction is now being considered as a manifestation of paucibacillary mycobacterial infection within the pleural space. With use of advanced culture media it’s possible to grow Mycobacterium tuberculosis from pleural fluid or tissue in approx 70 % of cases [4].

Microscopy for AFB in the pleural fluid can identify M. tuberculosis in fewer than 10 % of cases. The exception to this is patients with HIV and tuberculous empyema, where yields may be higher (>20 %). Our patient was HIV negative and had empyema which was positive for AFB from a revised national tuberculosis control program accredited laboratory.

Venous thrombosis is a rare complication which has been reported in 1.5–3.4 % of TB infections. There have been isolated case reports with involvement of hepatic veins [5], the vena porta, [6], the inferior vena cava [7], cerebral venous sinuses [8, 9], and the central retinal vein [10]. Mechanism behind hypercoagulability are decreased antithrombin lll and protein C, elevated plasma fibrinogen level, increased platelet aggregation and reactive thrombocytosis [10, 11]. Cytokines by virtue of their proinflammatory properties make vascular initima atherogenic and promote thrombosis. Apart from this high incidence of antiphospholipid antibodies detected in tuberculosis may also have a role [11].

Our patient had extrapulmonary tuberculosis with no evidence of pulmonary tuberculosis with venous thrombosis involving superior and inferior vena cava, right internal jugular vein and bilateral brachiocephalic vein. He also developed chylothorax, ascites with omental thickening with diffuse wall thickening of small bowel. Although these findings are consistent with tuberculosis but considered nonspecific as these may be seen in lymphoma, various forms of peritonitis, peritoneal carcinomatosis, and peritoneal mesothelioma. Association of pleural mesothelioma and tuberculosis has also been reported [12, 13]. Malignant mesothelioma has not been reported as a late sequelae of tuberculosis but its association with pleural mesothelioma has been reported [14].

Peritoneal mesothelioma is a rare, aggressive tumor with a median survival of 6–12 months. It’s seen more commonly in males, 50–69 years age group being most affected and major risk factor being exposure to crocidolite variety of asbestos [15]. Venous thrombosis in patients with peritoneal mesothelioma is very rare and the exact mechanism for the same is still a mystery. Although a few rare cases of pleural mesothelioma with venous thrombosis has been reported. Association of pleural mesothelioma and tuberculosis has also been reported. However, hypercoagulability associated with malignant disease is well recognized and causes predominantly venous thrombosis [16]. Usually cancer patients are prone to develop thrombosis either as paraneoplastic phenomena or due to hypercoagulable state caused by reduced protein C, protein S, antithrombin III and increased cytokines [17].

Our patient had peritoneal mesothelioma on biopsy but there was no asbestos exposure. Chylous pleural discharge may be explained by tumour invasion and compression of thoracic duct. Malignancy has been reported as the leading cause of nontraumatic chylothorax [18]. Tuberculosis may also cause chylothorax by enlarged lymphnodes compressing the thoracic duct. Multiple venous thromboses seen in our patient couldn’t be ascertained to a single pathology as both mesothelioma and tuberculosis have been shown to have a causal relationship with the same.

To the best of our knowledge there are no reported cases of tuberculosis coexisting with malignant peritoneal mesothelioma leading to multiple site venous thrombosis.

## Conclusions

In a case of tuberculosis not responding to treatment and having an uncommon presentation, possibility of underlying different pathology must be explored.

## Abbreviations

TB: tuberculosis
ATT: anti-tuberculosis therapy
CT: computed tomography
ICD: intercostal drainage
AFB: acid fast bacilli

## References

[1] Prapruttam D, Hedgire SS, Mani SE, Chandramohan A, Shyamkumar NK, Harisinghani M (2014). Tuberculosis—the great mimicker. Semin Ultrasound CT MRI.

[2] Udwadia ZF, Mehra C (2015). Tuberculosis in India. BMJ.

[3] Herzog BH (1998). History of tuberculosis. Respiration.

[4] Ruan S, Chuang Y, Wang J, Lin J, Chien J, Huang C (2012). Revisiting tuberculous pleurisy: pleural fluid characteristics and diagnostic yield of mycobacterial culture in an endemic area. Thorax.

[5] Gogna A, Grover S, Arun A, Saluja S (2004). Isolated hepatic inferior vena cava thrombosis in a case of tuberculosis—case report. J Indian Acad Clin Med.

[6] Ozşeker B, Ozşeker HS, Kav T, Shorbagi A, Karakoç D, Bayraktar Y (2012). Abdominal tuberculosis leading to portal vein thrombosis, mimicking peritoneal carcinomatosis and liver cirrhosis. Acta Clin Belg.

[7] Raj M, Agrawal A (2006). Inferieur vena cava thrombosis complicating tuberculosis. N.Z. Med. J.

[8] Fiorot J, Felício A, Fukujima M, Rodrigues C, Morelli V, Lourenço D (2005). Tuberculosis: an uncommon cause of cerebral venous thrombosis?. Arq Neuropsiquiatr.

[9] Sundaram PK, Sayed F (2007). Superior sagittal sinus thrombosis caused by calvarial tuberculosis: case report. Neurosurgery.

[10] Fullerton DG, Shrivastava A, Munavvar M, Jain S, Howells J, Macdowall P (2007). Pulmonary tuberculosis presenting with central retinal vein occlusion. Br J Ophthalmol.

[11] Mark PL, Ashok PP, Deshpande RB, Mahashur AA (2009). A patient with hypercoagulable state due to tuberculosis. Indian J Chest Dis Allied Sci.

[12] Smiti S, Rajagopal KV (2010). CT mimics of peritoneal carcinomatosis. Indian J Radiol Imaging.

[13] Epstein BM, Mann JH (1982). CT of abdominal tuberculosis. Am J Roentgenol.

[14] Roviaro GC, Sartori F, Calabrò F, Varoli F (1982). The association of pleural mesothelioma and tuberculosis. Am Rev Respir Dis.

[15] Cunha P, Luz Z, Seves I, Sousa C, Skiappa Ribeiro L (2002). Malignant peritoneal mesothelioma—diagnostic and therapeutic difficulties. Acta Med Port.

[16] Schattner A, Kozack N (2004). A 47-year-old man with mesothelioma and neck swelling. Can Med Assoc J.

[17] Bridda A, Padoan I, Mencarelli R, Frego M (2007). Peritoneal mesothelioma: a review. Medscape Gen Med.

[18] Doerr CH, Allen MS, Nichols FC, Ryu JH (2005). Etiology of chylothorax in 203 patients. Mayo Clin Proc.

